# Nomogram for predicting the overall survival and cancer-specific survival of patients with extremity liposarcoma: a population-based study

**DOI:** 10.1186/s12885-020-07396-x

**Published:** 2020-09-16

**Authors:** Lin Ye, Chuan Hu, Cailin Wang, Weiyang Yu, Feijun Liu, Zhenzhong Chen

**Affiliations:** 1 Department of Orthopedics, 5th Affiliated Hospital, Lishui Municipal Central Hospital, Wenzhou Medical College, Lishui, 323000 Zhejiang China; 2 Medical college, Qingdao University, Qingdao, 266071 Shandong China; 3 Wenzhou Medical College, Wenzhou, 325000 Zhejiang China

**Keywords:** Extremity, Liposarcoma, Nomogram, Overall survival, Cancer-specific survival

## Abstract

**Background:**

Extremity liposarcoma represents 25% of extremity soft tissue sarcoma and has a better prognosis than liposarcoma occurring in other anatomic sites. The purpose of this study was to develop two nomograms for predicting the overall survival (OS) and cancer-specific survival (CSS) of patients with extremity liposarcoma.

**Methods:**

A total of 2170 patients diagnosed with primary extremity liposarcoma between 2004 and 2015 were extracted from the Surveillance, Epidemiology, and End Results (SEER) database. Univariate and multivariate Cox analyses were performed to explore the independent prognostic factors and establish two nomograms. The area under the curve (AUC), C-index, calibration curve, decision curve analysis (DCA), Kaplan-Meier analysis, and subgroup analyses were used to evaluate the nomograms.

**Results:**

Six variables were identified as independent prognostic factors for both OS and CSS. In the training cohort, the AUCs of the OS nomogram were 0.842, 0.841, and 0.823 for predicting 3-, 5-, and 8-year OS, respectively, while the AUCs of the CSS nomogram were 0.889, 0.884, and 0.859 for predicting 3-, 5-, and 8-year CSS, respectively. Calibration plots and DCA revealed that the nomogram had a satisfactory ability to predict OS and CSS. The above results were also observed in the validation cohort. In addition, the C-indices of both nomograms were significantly higher than those of all independent prognostic factors in both the training and validation cohorts. Stratification of the patients into high- and low-risk groups highlighted the differences in prognosis between the two groups in the training and validation cohorts.

**Conclusion:**

Age, sex, tumor size, grade, M stage, and surgery status were confirmed as independent prognostic variables for both OS and CSS in extremity liposarcoma patients. Two nomograms based on the above variables were established to provide more accurate individual survival predictions for extremity liposarcoma patients and to help physicians make appropriate clinical decisions.

## Background

Liposarcoma is a rare malignant tumor accounting for approximately 15 to 20% of soft tissue sarcoma (STS) [1]. It is estimated that 13,130 new cases of STS and 5350 deaths due to STS will occur in the United States in 2020 [2]. Liposarcoma can occur in any site but is usually located in the retroperitoneum and extremities [3]. Extremity liposarcoma represents 25% of extremity STS and has a better prognosis than that liposarcoma other locations [4, 5]. Currently, surgical resection with adjuvant radiation therapy is one of the main treatment strategies for extremity STS patients [6]. In addition, chemotherapy may also be considered for patients with localized disease but at high risk of developing distant metastasis and patients with metastatic disease amenable to surgery at the initial diagnosis [7–9].

Currently, the American Joint Committee on Cancer (AJCC) system, known as the TNM staging system, remains the gold standard for prognostic prediction for tumor patients. However, other elements that have been reported to be prognostic factors for extremity STS patients are not taken into consideration in the TNM staging system, such as patient factors (including age and sex), tumor characteristics (including tumor grade, histologic subtype, and tumor location), and treatment strategies (including surgery, radiotherapy, and chemotherapy) [7, 9–15]. More importantly, the TNM staging system is unable to meet the increasing need for precision medicine and cannot provide individual predictions of prognosis at specific times [16, 17].

Considering the various clinicopathologic characteristics that could affect the prognosis of patients with extremity liposarcoma, an instrument integrating the relevant prognostic predictors is urgently needed to facilitate therapeutic invention and enhance patient quality of life. The nomogram is a pictorial representation of a multivariable model in which the relative contribution of each covariate on the outcome of interest is considered, and nomograms are a practical tool in oncology and medicine [3, 18–20]. However, no extremity liposarcoma-specific nomogram has been established for estimating individual patient outcomes by integrating all relevant predictors.

Based on the Surveillance, Epidemiology, and End Results (SEER) program database, this study aimed to identify the prognostic factors of extremity liposarcoma patients and develop two nomograms to predict overall survival (OS) and cancer-specific survival (CSS).

## Methods

### Patients

We identified all patients with primary extremity liposarcoma between 2004 and 2015 with SEER Stat 8.3.6, which was publicly available and did not include personal information. The inclusion criteria were as follows: (1) confirmed histologic type of liposarcoma; (2) site limited to the extremities; (3) primary tumor; (4) age at diagnosis ≥18 years; and (5) known cause of death and complete follow-up data. The exclusion criteria were as follows: (1) unknown age, sex, AJCC TNM status, tumor size, tumor grade, histologic subtype, cause of death, and follow-up time; (2) local recurrence or distal metastatic tumors after treatment; and (3) survival time < 1 month.

Patients who met the abovementioned criteria were randomly divided into the training set (70%) and testing set (30%). In our study, the nomograms were established based on the training set and were validated in the testing set.

### Variables

The variables utilized in the current study were age at diagnosis, race, sex, histologic subtype, tumor size, tumor grade, AJCC stage, T stage, N stage, M stage, surgery information, radiotherapy information, and chemotherapy data. Age and tumor size were translated into categorical variables, and the cutoff values were calculated by X-tile software [21]. In this software, all possible divisions of the marker data are assessed and a χ2 value is calculated for every possible division of the population [21]. Then, this program can select the optimal division of the data by selecting the highest χ2 value [21]. AJCC stage was categorized as stage I/II and stage III/IV. T stage was divided into T1 and T2. N stage and M stage were described as either negative or positive. Tumor grade was classified as well differentiated, moderately differentiated, poorly differentiated, and undifferentiated anaplastic. In the present study, OS and CSS were considered as the outcomes. OS was defined as the interval from the date of the primary diagnosis to the date of death due to any cause. CSS was defined as the interval from the date of the primary diagnosis to the date of liposarcoma-specific death.

### Statistical analysis

The optimal cutoff values for tumor size and age at diagnosis were separately confirmed using X-tile software based on OS and CSS information. Univariate and multivariate Cox analyses were performed to explore the independent prognostic factors for OS and CSS. Based on the multivariable Cox regression models, two nomograms for 3-, 5-, and 8-year OS and CSS were constructed. The C-indices of the proposed nomograms and each single independent factor were calculated, and a comparison of the C-indices was performed to assess the discrimination of the nomogram with the CsChange package. In addition, the time-dependent receiver operating characteristic (ROC) curves for the models were established, and the areas under the curves (AUCs) were computed to show the discrimination of the nomograms for 3-, 5-, and 8-year OS and CSS. Calibration curves were also established to compare the nomogram-predicted probability with the observed outcome, and decision curve analysis (DCA) was used to show the clinical utilization of the nomogram. Finally, we further categorized patients into high- and low-risk groups according to their median risk score. Survival analysis was then performed with the Kaplan–Meier method to probe the differences in prognosis between the two risk groups, and the log-rank test was performed. In the present study, all statistical analyses were performed with SPSS 25.0, and a *p* value< 0.05 (two-sided) was considered statistically significant. The nomograms, C-indices, ROC curves, calibration curves, DCA analyses, and Kaplan–Meier curves were generated with R software (version 3.6.1).

## Results

### Baseline patient demographics

In our study, 2170 patients with extremity liposarcoma who met the criteria were included and were divided into the training (*n* = 1522) and validation cohorts (*n* = 648). The baseline demographics and clinicopathologic characteristics are listed in Table 1. The optimal cutoff values of tumor size and age were identified separately based on OS and CSS (Fig. S1). Tumor size was divided into < 11.1 cm, 11.1–23.5 cm, and > 23.5 cm based on OS information, while it was grouped as < 7.4 cm, 7.4–12.4 cm, and > 12.4 cm based on CSS information (Fig. S1B and D). Moreover, the optimal cutoff values of age were identified as 65 and 76 years based on OS status, and the same cutoff ages were identified based on CSS status (Fig. S1A and C).

**Table 1:** Baseline of extremity liposarcoma patients

	Training cohort	Validation cohort
Age, year	55.53 ± 16.47	55.21 ± 16.53
Tumor size, cm	13.75 ± 8.48	13.53 ± 8.19
Race		
Black	165	76
Other	131	51
White	1226	521
Sex		
Female	646	284
Male	876	364
Histological type		
Liposarcoma, NOS	181	73
Liposarcoma, well differentiated	538	230
Myxoid liposarcoma	443	198
Round cell liposarcoma	46	24
Pleomorphic liposarcoma	131	60
Mixed liposarcoma	62	27
Fibroblastic liposarcoma	2	2
Dedifferentiated liposarcoma	119	34
AJCC		
I/II	1229	541
III/IV	293	107
T		
T1	213	101
T2	1309	547
N		
N0	1516	645
N1	6	3
M		
M0	1498	628
M1	24	20
Surgery performed	1494	635
Radiotherapy performed	714	294
Chemotherapy performed	165	65
Primary site		
Lower extremity	1333	557
Upper extremity	189	91
Grade		
I	829	358
II	286	135
III	196	75
IV	211	80

### Identification of prognostic factors

The results of the univariate analyses in the training cohort are shown in Table 2 and Table 3. The significant variables for OS were age, sex, tumor grade, certain histologic subtypes, tumor size, AJCC stage, M stage, surgery, chemotherapy, and radiotherapy. In addition to the above ten factors, T stage was statistically associated with CSS. These factors were further included the multivariate Cox analysis. Finally, age, sex, tumor size, AJCC stage, M stage, and surgery were identified as independent prognostic predictors for both OS and CSS (Table 2 and Table 3).

**Table 2:** Survival analyses of overall survival for extremity liposarcoma patients

	Univariate analysis	Multivariate analysis			
	P	HR	95.0% CI		P
Age					
< 65	Reference	Reference			
65–76	< 0.001	1.91	1.41–2.58		< 0.001
> 76	< 0.001	5.64	4.23–7.53		< 0.001
Tumor size					
< 11.1	Reference	Reference			
11.1–23.5	0.022	1.69	1.29–2.22		< 0.001
> 23.5	< 0.001	2.52	1.77–3.57		< 0.001
Race					
Black	Reference				
Other	0.426				
White	0.668				
Sex					
Female	Reference	Reference			
Male	0.002	1.43	1.11–1.84		0.006
Histological type					
Liposarcoma, NOS	Reference				
Liposarcoma, well differentiated	0.001				
Myxoid liposarcoma	0.327				
Round cell liposarcoma	0.005				
Pleomorphic liposarcoma	< 0.001				
Mixed liposarcoma	0.256				
Fibroblastic liposarcoma	0.951				
Dedifferentiated liposarcoma	< 0.001				
AJCC					
I/II	Reference				
III/IV	< 0.001				
T					
T1	Reference				
T2	0.273				
N					
N0	Reference				
N1	0.063				
M					
M0	Reference	Reference			
M1	< 0.001	4.97	2.92–8.46		< 0.001
Surgery					
No	Reference	Reference			
Yes	< 0.001	0.33	0.20–0.55		< 0.001
Radiotherapy					
No	Reference				
Yes	< 0.001				
Chemotherapy					
No	Reference				
Yes	< 0.001				
Primary site					
Lower extremity	Reference				
Upper extremity	0.730				
Grade					
I	Reference	Reference			
II	0.312	1.83	1.22–2.74		0.004
III	< 0.001	4.90	3.53–6.80		< 0.001
IV	< 0.001	5.85	4.27–8.02		< 0.001

*HR* Hazard ratio, *CI* Confidence interval, *AJCC* American Joint Committee on Cancer

**Table 3:** Survival analyses of cancer-specific survival for extremity liposarcoma patients

	Univariate analysis	Multivariate analysis			
	P	HR	95.0% CI		P
Age					
< 65	Reference	Reference			
65–76	0.416	1.25	0.83–1.88		0.277
> 76	< 0.001	3.26	2.19–4.85		< 0.001
Tumor size					
< 7.4	Reference	Reference			
7.4–12.4	0.036	2.42	1.43–4.10		0.001
> 12.4	< 0.001	3.73	2.32–6.01		< 0.001
Race					
Black	Reference				
Other	0.609				
White	0.668				
Sex					
Female	Reference	Reference			
Male	0.001	1.42	1.01–2.00		0.047
Histological type					
Liposarcoma, NOS	Reference				
Liposarcoma, well differentiated	< 0.001				
Myxoid liposarcoma	0.668				
Round cell liposarcoma	< 0.001				
Pleomorphic liposarcoma	< 0.001				
Mixed liposarcoma	0.024				
Fibroblastic liposarcoma	0.966				
Dedifferentiated liposarcoma	0.002				
AJCC					
I/II	Reference				
III/IV	< 0.001				
T					
T1	Reference				
T2	0.007				
N					
N0	Reference				
N1	0.088				
M					
M0	Reference	Reference			
M1	< 0.001	5.83	3.37–10.09		< 0.001
Surgery					
No	Reference	Reference			
Yes	< 0.001	0.43	0.21–0.85		0.015
Radiotherapy					
No	Reference				
Yes	< 0.001				
Chemotherapy					
No	Reference				
Yes	< 0.001				
Primary site					
Lower extremity	Reference				
Upper extremity	0.768				
Grade					
I	Reference	Reference			
II	< 0.001	3.95	2.13–7.33		< 0.001
III	< 0.001	14.10	8.34–23.85		< 0.001
IV	< 0.001	19.02	11.41–31.71		< 0.001

*HR* Hazard ratio, *CI* Confidence interval, *AJCC* American Joint Committee on Cancer

### Construction of the prognostic nomograms

**Fig. 1:**
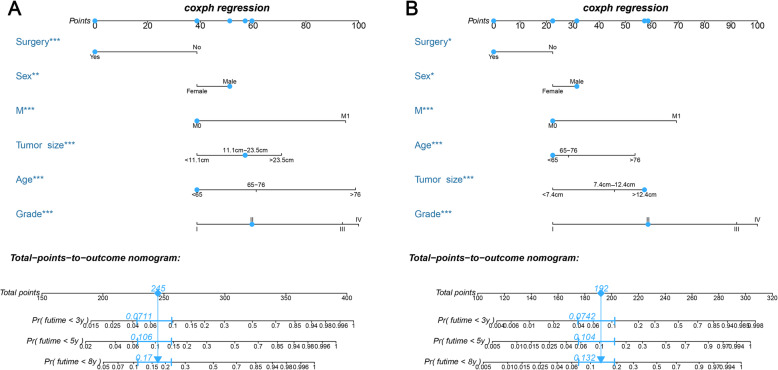
**a** A nomogram to predict 3-, 5-, and 8-year OS for extremity liposarcoma patients; **b** A nomogram to predict 3-, 5-, and 8-year CSS for extremity liposarcoma patients. The blue example shows how to use the nomogram. OS: overall survival; CSS: cancer-specific survival

**Fig. 2:**
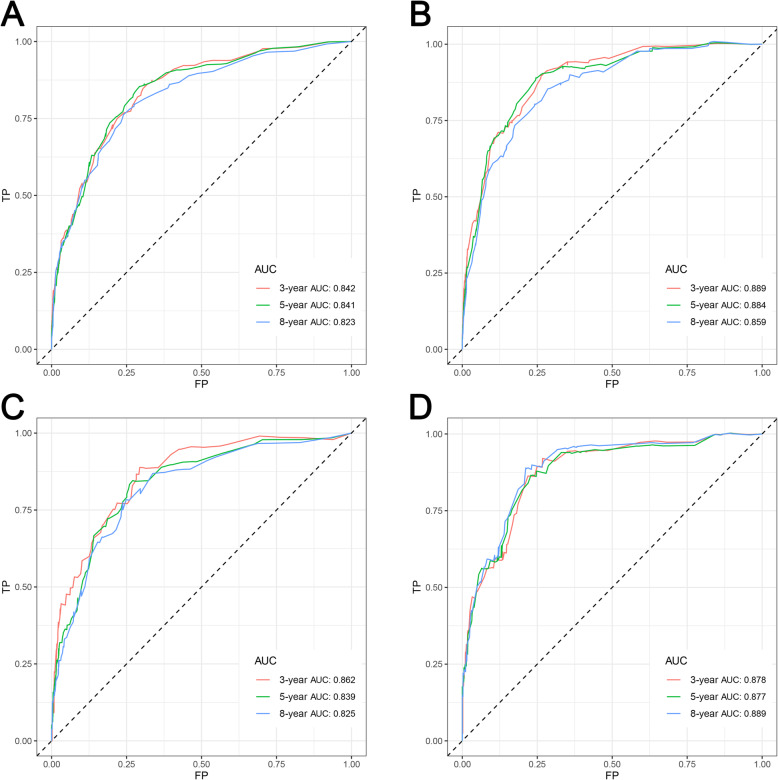
Time-dependent ROC curves. **a** Time-dependent ROC curves of the OS nomogram showed that the AUCs in the training cohort were 0.842, 0.841, and 0.823 for predicting 3-, 5-, and 8-year OS, respectively; **b** Time-dependent ROC curves of the CSS nomogram in the training cohort showed that the AUCs were 0.889, 0.884, and 0.859 for predicting 3-, 5-, and 8-year CSS, respectively; **c** Time-dependent ROC curves of the OS nomogram showed that the AUCs in the validation cohort were 0.862, 0.839, and 0.825 for predicting 3-, 5-, and 8-year OS, respectively; **d** Time-dependent ROC curves of the CSS nomogram in the validation cohort showed that the AUCs were 0.878, 0.877, and 0.889 for predicting at 3-, 5-, and 8-year CSS, respectively. ROC: receiver operating characteristic; AUC: area under the curve; OS: overall survival; CSS: cancer-specific survival

**Fig. 3:**
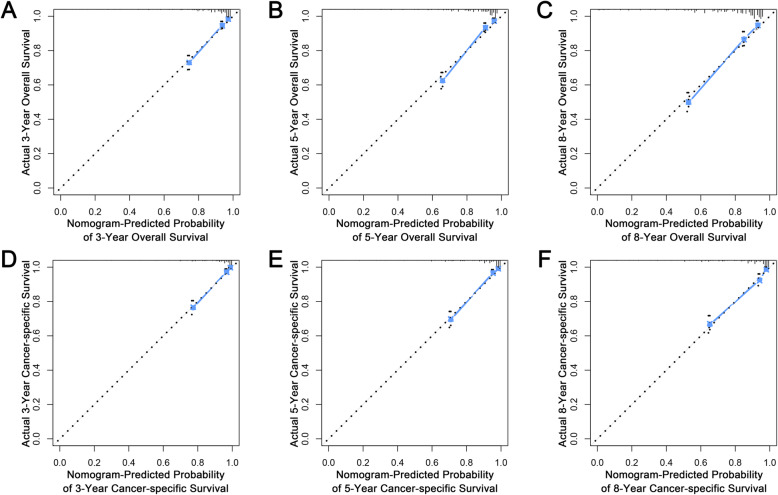
Calibration curves in the training cohort. **a-c** Calibration curves of the OS nomogram for predicting 3-, 5-, and 8-year OS; **d-f** Calibration curves of the CSS nomogram for predicting 3-, 5-, and 8-year CSS. OS: overall survival; CSS: cancer-specific survival

Based on the multivariate Cox model, two nomograms that integrated the aforementioned significant independent predictors are demonstrated in Fig. 1a and b. With these nomograms, we can obtain the corresponding survival probability of each patient by adding up the specific points of each predictor. The ROC curves demonstrated the good discriminative abilities of the nomograms (Fig. 2a and b). The AUCs of the nomogram for predicting 3-, 5-, and 8-year OS were 0.842, 0.841, and 0.823, respectively. The AUCs of the nomogram for predicting 3-, 5-, and 8-year CSS were 0.889, 0.884, and 0.859, respectively. The calibration curves of OS (Fig. 3a-c) and CSS (Fig. 3d-f) showed optimal agreement between the predicted and observed survival probabilities. Moreover, DCA showed that both nomograms have favorable clinical utilization (Fig. S2A-F).

### Validation of the nomograms in the validation set

**Fig. 4:**
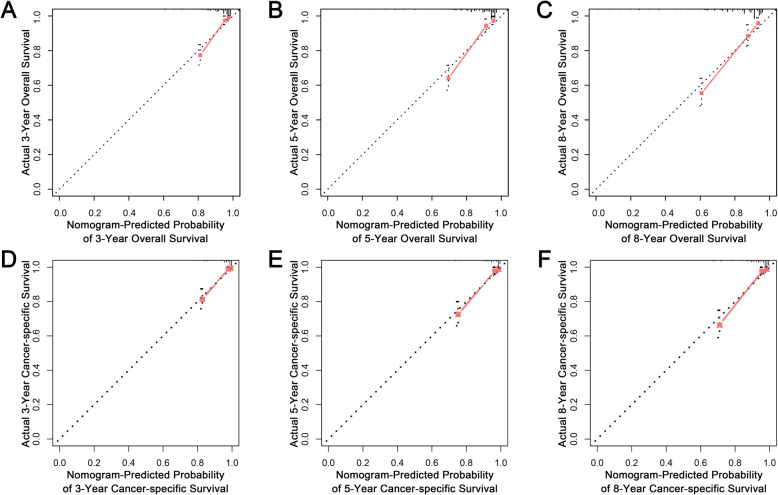
Calibration curves in the validation cohort. **a-c** Calibration curves of the OS nomogram for predicting at 3-, 5-, and 8-year OS; **d-f** Calibration curves of the CSS nomogram for predicting 3-, 5-, and 8-year CSS. OS: overall survival; CSS: cancer-specific survival

The performance of the nomogram in the external validation set also showed favorable outcomes. The AUC values of the nomogram for predicting 3-, 5-, and 8-year OS were 0.862, 0.839, and 0.825, respectively (Fig. 2c). The AUC values of the nomogram for predicting 3-, 5-, and 8-year CSS were 0.878, 0.877, and 0.889, respectively (Fig. 2d). The calibration curves for the OS (Fig. 4a-c) and CSS (Fig. 4d-f) probabilities further validated the nomograms. More importantly, favorable clinical utilization of the nomograms was also confirmed in the validation cohort (Fig. S3A-F).

### Comparison of discrimination between the nomograms and single factors

**Fig. 5:**
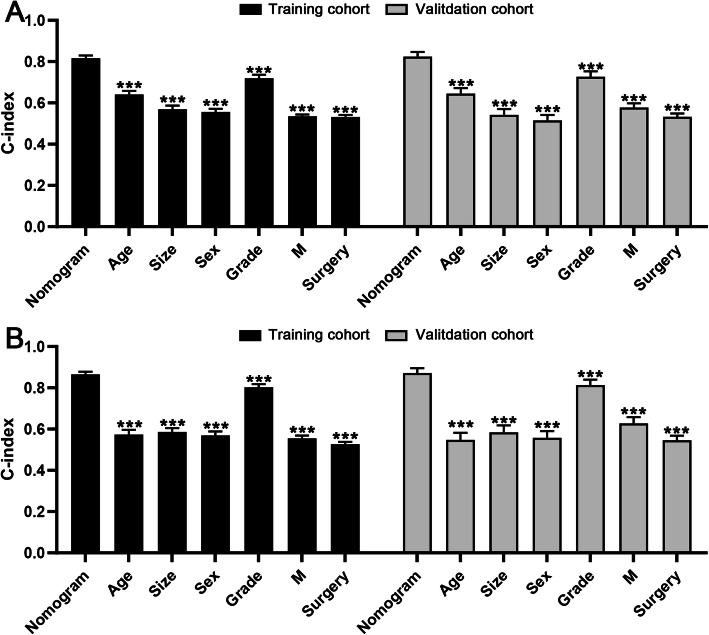
Comparison of C-indices between the nomograms and single factors. **a** The C-index of the OS nomogram was significantly higher than that of the six independent prognostic factors, in both the training cohort and validation cohort; **b** The C-index of the CSS nomogram was significantly higher than that of the six independent prognostic factors, in both the training cohort and validation cohort. OS: overall survival; CSS: cancer-specific survival

In the current study, age, sex, tumor size, AJCC staging, M stage, and surgery were confirmed as independent prognostic factors for extremity liposarcoma. Two nomograms based on the above variables were constructed and validated. The predictive power of the proposed nomograms and each single independent factor was assessed by the C-index. As shown in Fig. 5a, the C-index of the OS nomogram was significantly higher than that of the indices of age, sex, tumor grade, M status, and surgery status (*P* < 0.001), in both the training and validation cohorts. Moreover, the C-index of the CSS nomogram was also superior to that of single independent factors in both the training and validation cohorts (*P* < 0.001) (Fig. 5b).

### Risk stratification for extremity liposarcoma patients

**Fig. 6:**
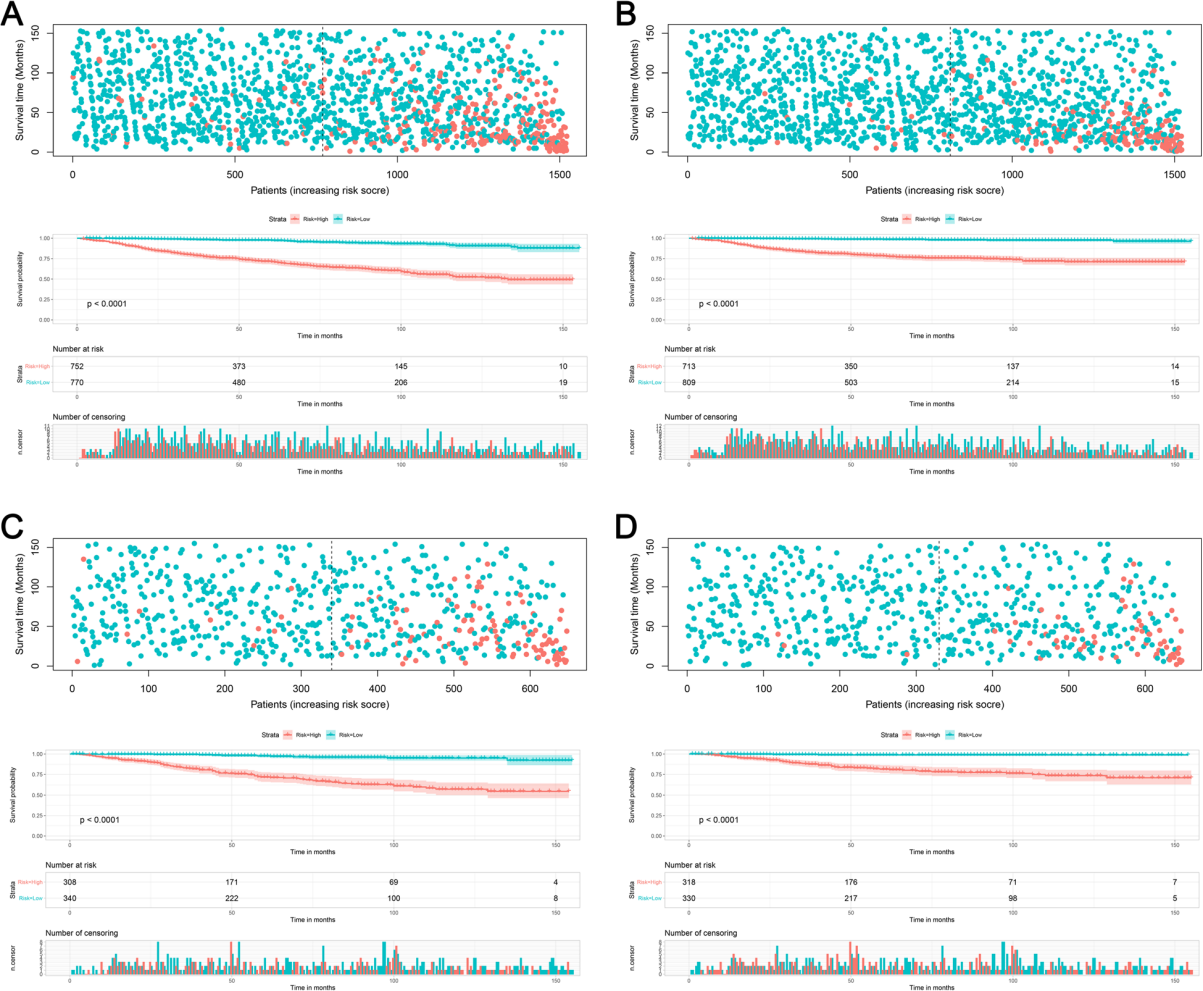
Kaplan–Meier survival analyses for all patients according to our risk stratification. Survival curves showed the OS (**a**) and CSS (**b**) of the high-risk (red) and low-risk (blue) groups in the training cohort and the OS (**c**) and CSS (**d**) in the validation cohort. OS: overall survival; CSS: cancer-specific survival

Risk stratification is very important for guiding patient management. Therefore, we further stratified the patients into high- and low-risk groups according to their median of risk score. Kaplan–Meier survival analysis showed favorable OS and CSS in the low-risk group compared with the high-risk group (Fig. 6a and b). In the validation cohort, a favorable prognosis was also observed in the low-risk group, for both OS and CSS (Fig. 6c and d).

## Discussion

In the present study, older age, male sex, higher tumor grade, larger tumor size, absence of surgery, and distant metastasis were found to be risk factors for both worse OS and CSS in extremity liposarcoma patients. We then developed and validated extremity liposarcoma nomograms to estimate 3-, 5-, and 8-year OS and CSS. Discrimination, calibration and clinical utilization analyses were employed to evaluate the performance of these nomograms as predictive tools, and these results confirmed that our nomograms were effective and accurate models. The proposed nomograms also showed a good ability to categorize patients into high-risk and low-risk groups with significant differences in OS and CSS.

Compared with the previous nomogram from MSKCC [3], our nomograms have several improvements. First, the MSKCC nomogram for all liposarcoma patients was developed based on a cohort from a single institution, and there were only 452 extremity liposarcoma patients. In contrast, our nomograms were developed based on a population-based cohort of 1522 patients and validated in 648 patients, allowing us to develop extremity liposarcoma-specific nomograms. Second, the MSKCC nomogram included postoperative variables, making it an inadequate preoperative counseling tool. This limitation no longer exists in our nomograms, which means that the prognosis of patients with extremity liposarcoma can be accurately predicted preoperatively. Finally, our nomograms were developed in the training cohort and validated in the validation cohort. ROC curves, C-indices, calibration curves, and DCAs were used to evaluate the performance of the nomograms. Such a comprehensive analysis is also an important improvement in our research.

We categorized patients into three groups by identifying 65 and 76 as optimal age cutoffs via X-tile software. Our results showed that increasing age was associated with a worse survival outcome. A previous study on liposarcoma also reported that age was an independent prognostic predictor [3]; conversely, no clear association between age and survival was observed in a retrospective evaluation over 15 years [3]. Further studies demonstrated that younger patients were more likely to be diagnosed with smaller tumors (≤5 cm vs > 5 cm) [22], distal extremity STS (distal extremities vs other limb localizations) [10], and only pulmonary metastases (pulmonary lesions vs other lesions) [7], and these patients tended to be more easily cured and therefore had a better prognosis. Similarly, a distribution difference by age in terms of tumor location and metastatic sites was detected [7, 10], which may also explain why males were associated with unfavorable outcomes. Nevertheless, whether there was a biological reason behind these age and sex distributions is unclear.

Previous studies have identified large tumor size as an indicator of poor prognosis for extremity STS patients [3, 10, 13, 14, 22], consistent with the present research. This is probably because a large tumor size is related to higher biologic malignancy, including regional invasiveness and metastatic potential. It was also true that more complex and radical surgery was considered for patients with large masses, resulting in poor quality of life. Tumor grade was proven to be an important prognostic predictor of extremity liposarcoma in our study. Previous studies also revealed that tumor grade was significantly associated with metastatic potential after surgery and therefore risk of death. However, tumor grade had poor value in predicting local recurrence, which was mainly correlated with suboptimal surgical procedures [10, 23, 24]. In clinical practice, patients with high-grade tumors or tumors with large diameters were selected for combination therapy with neoadjuvant chemotherapy to limit the risk of distant metastases [8].

Regional lymphatic spread of extremity liposarcoma has not been discussed. In the present study, there was no significant difference in survival between patients with N0 (node negative) and N1 (node positive) disease, suggesting that extremity liposarcoma were more likely to develop hematogenous metastasis than lymphatic metastasis, similar to most STSs [25]. Ethun et al. reported that lymphovascular invasion, which was defined as the presence of tumor cells within the lumen of either lymph or blood vessels on hematoxylin-eosin (H&E) staining, was an important adverse pathologic factor in truncal and extremity STS [26]. However, the author did not analyze lymph invasion and vascular invasion separately. A further prospective study should be performed to study the impact of lymph invasion on patient outcomes. Patients usually die of distant metastasis identified at the initial diagnosis or after surgery, suggesting that the presence of systemic disease rather than the primary tumor drove the outcomes [7, 11, 14, 25, 27]. Consistent with previous studies, we found that patients who developed metastatic disease had a worse prognosis. Lung metastases are most commonly associated with favorable outcomes [7]. However, liposarcoma also has an unusual propensity to metastasize to the retroperitoneum, mediastinum, and bone, which are seldomly amenable to curative treatment [11, 25, 27].

Surgical resection remains the cornerstone of treatment for extremity liposarcoma. Before the 1970s, amputation was the main therapeutic method, which led to decreased recurrence but increased disabilities compared with pure local excision [28]. Currently, the combination of wide excision and preoperative radiation therapy is widely adopted as the primary treatment [9]. Despite the limited local recurrence with adjuvant radiation or margin-negative resections with radical surgery, patients are still at risk of developing secondary metastases, which suggests that biological aggression is the primary determinant of patient outcome [8, 10, 28]. Considering this, there has been growing utilization of chemotherapy for patients with extremity STS, especially myxoid liposarcoma, which is relatively chemosensitive [29, 30]. Although chemotherapy led to a decreased incidence of metastasis after surgery and benefits in metastatic patients, whether this treatment provided prolonged OS was unclear [7, 9, 31]. Because of the large amount of unknown information, our result was underpowered to clarify the impact of radiotherapy and chemotherapy on survival.

Several limitations of this study need to be acknowledged. First, since this study is a retrospective study based on a large database, information and selection bias may have been introduced. Second, we did not take tumor location into account, while previous studies indicated that lower limb or distal extremity sarcomas were associated with reduced OS. Third, the SEER database does not provide access to detailed clinical information. Tumor depth, metastatic sites and operation methods that might have an impact on survival were not documented. Additionally, the detailed data regarding surgery, chemotherapy, and radiation therapy were incomplete, and the reason why some patients did not undergo surgery is unclear. Fourth, our nomograms can only predict OS and CSS to a maximum of 8 years due to the limited follow-up period. Despite these limitations, this was a large population-based study that investigated the prognostic factors of patients with extremity liposarcoma, and the favorable utilization of the nomograms was confirmed.

## Conclusion

The current study identified age, sex, tumor size, grade and metastasis as prognostic factors for both OS and CSS in patients with extremity liposarcoma. These factors were incorporated to construct the nomograms. The established nomograms may assist with patient counseling and help physicians make appropriate clinical decisions.

## Supplementary information

**Additional file 1: Fig. S1.** The results of X-tile software showing the best cutoff values of age and tumor size. (A) The best cutoff value of age based on the follow-up OS data; (B) The best cutoff value of tumor size based on the follow-up OS data; (C) The best cutoff value of age based on the follow-up CSS data; (D) The best cutoff value of tumor size based on the follow-up CSS data. OS: overall survival; CSS: cancer-specific survival.

**Additional file 2: Fig. S2.** Decision curve analyses in the training cohort. (A-C) Decision curve analyses of the OS nomogram for predicting 3-, 5-, and 8-year OS; (D-F) Decision curve analyses of the CSS nomogram for predicting at 3-, 5-, and 8-year CSS. OS: overall survival; CSS: cancer-specific survival.

**Additional file 3: Fig. S3.** Decision curve analyses in the validation cohort. (A-C) Decision curve analyses of the OS nomogram for predicting at 3-, 5-, and 8-year OS; (D-F) Decision curve analyses of the CSS nomogram for predicting at 3-, 5-, and 8-year CSS. OS: overall survival; CSS: cancer-specific survival.

## Data Availability

The dataset from SEER database generated and/or analyzed during the current study are available in the SEER dataset repository (https://seer.cancer.gov/).

## Abbreviations

OS: Overall survival
CSS: Cancer-specific survival
SEER: Surveillance, Epidemiology, and End Results
AUC: Area under the curve
ROC: Receiver operating characteristic
DCA: Decision curve analysis
STS: Soft tissue sarcoma
AJCC: American Joint Committee on Cancer
