# Supplementary figures and images for: Nomogram for predicting the overall survival and cancer-specific survival of patients with extremity liposarcoma: a population-based study
Source: BMC Cancer. 2020 Sep 16;20:889. doi: 10.1186/s12885-020-07396-x (PMC7493333; doi:10.1186/s12885-020-07396-x)

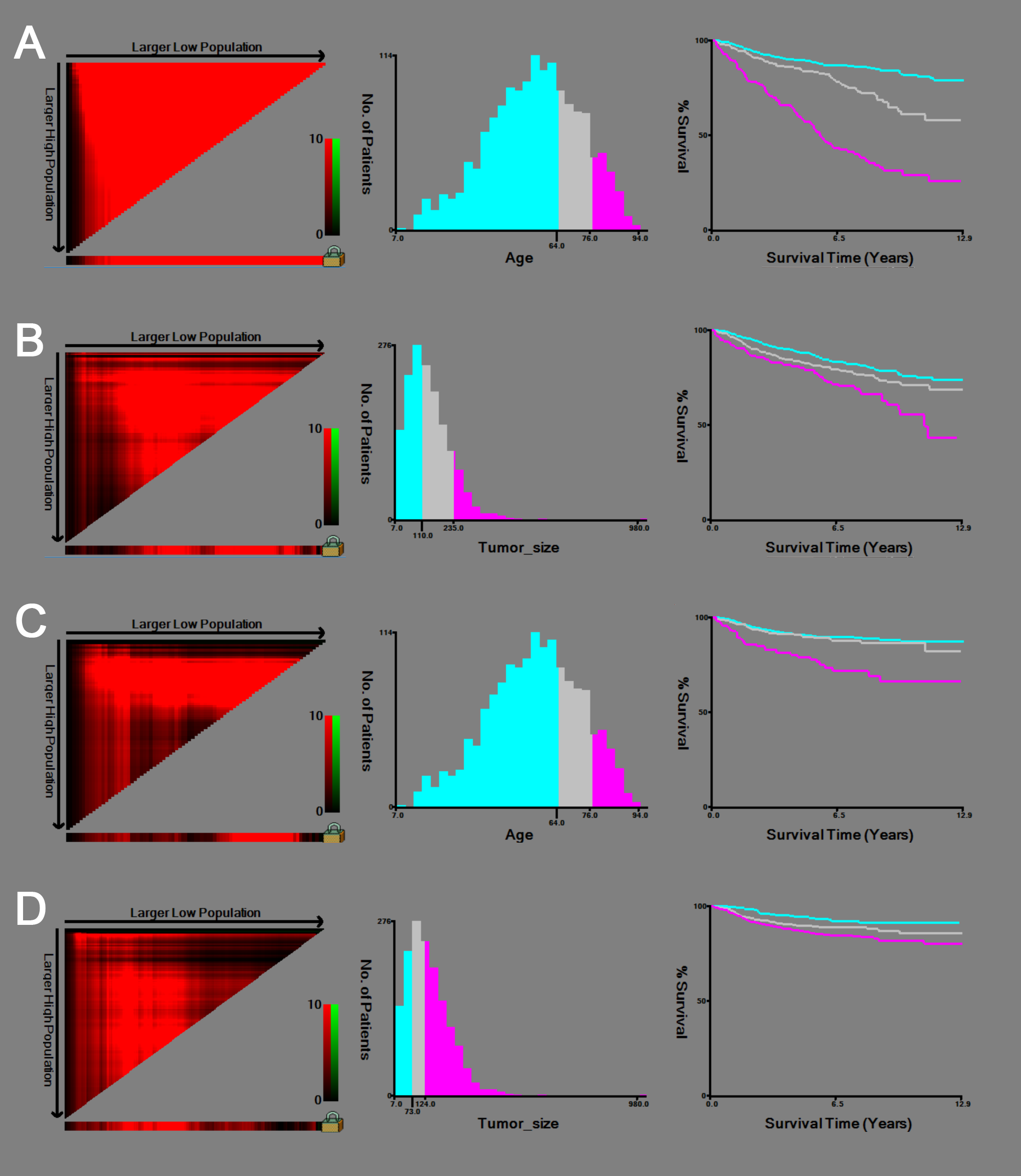

Supplement: Supplementary file 1 — Additional file 1: Fig. S1. The results of X-tile software showing the best cutoff values of age and tumor size. (A) The best cutoff value of age based on the follow-up OS data; (B) The best cutoff value of tumor size based on the follow-up OS data; (C) The best cutoff value of age based on the follow-up CSS data; (D) The best cutoff value of tumor size based on the follow-up CSS data. OS: overall survival; CSS: cancer-specific survival. [file 12885_2020_7396_MOESM1_ESM.tif]

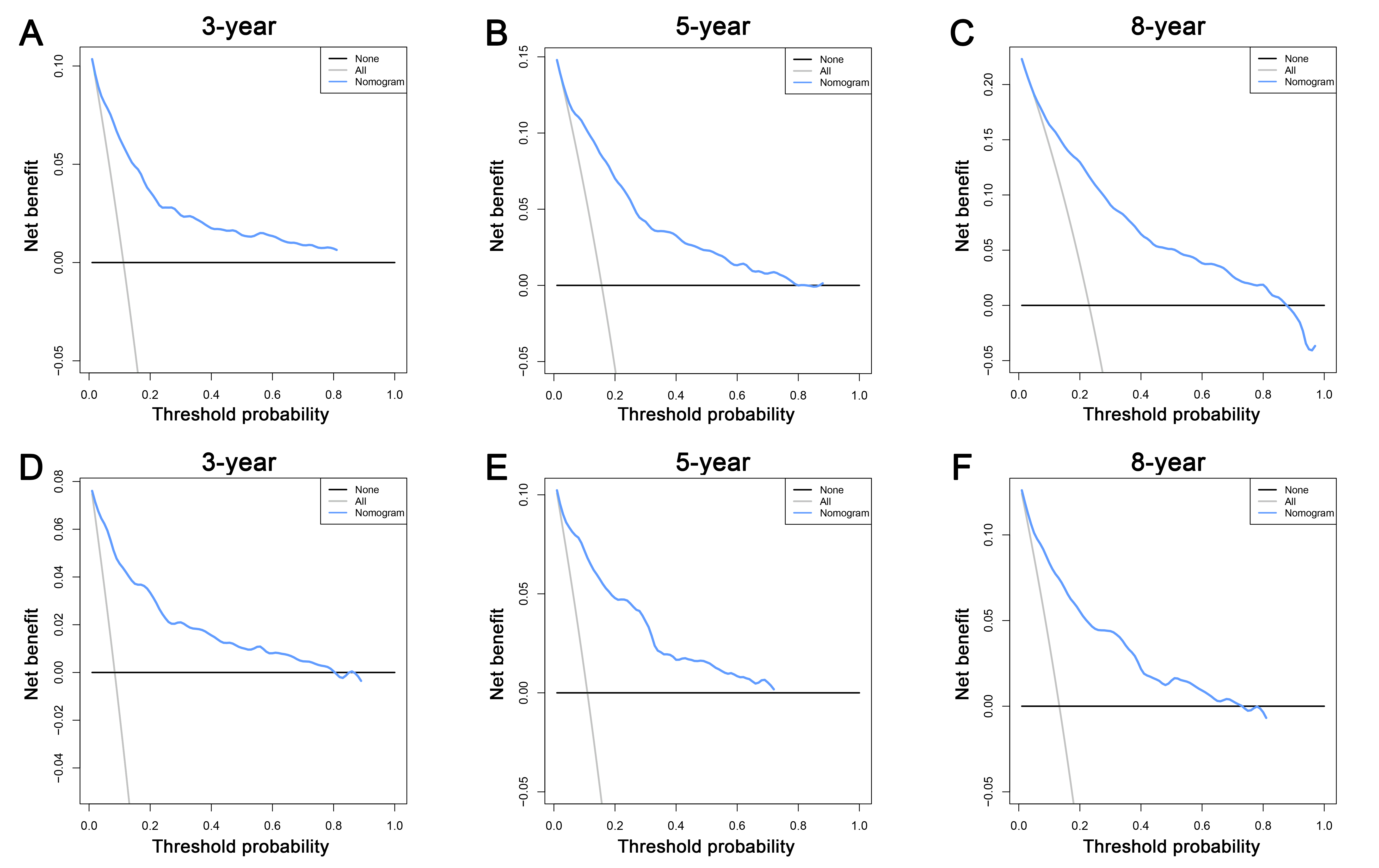

Supplement: Supplementary file 2 — Additional file 2: Fig. S2. Decision curve analyses in the training cohort. (A-C) Decision curve analyses of the OS nomogram for predicting 3-, 5-, and 8-year OS; (D-F) Decision curve analyses of the CSS nomogram for predicting at 3-, 5-, and 8-year CSS. OS: overall survival; CSS: cancer-specific survival. [file 12885_2020_7396_MOESM2_ESM.tif]

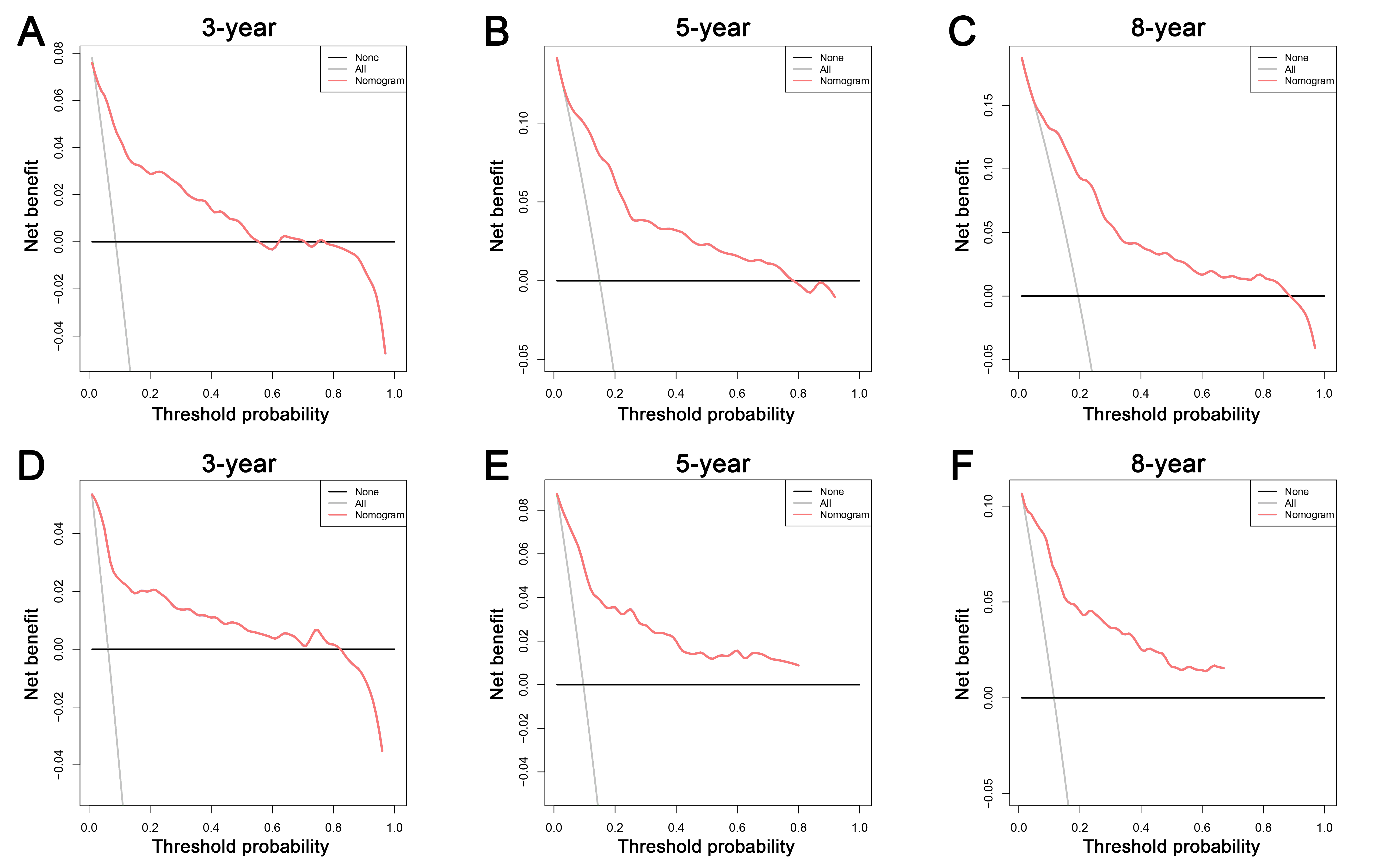

Supplement: Supplementary file 3 — Additional file 3: Fig. S3. Decision curve analyses in the validation cohort. (A-C) Decision curve analyses of the OS nomogram for predicting at 3-, 5-, and 8-year OS; (D-F) Decision curve analyses of the CSS nomogram for predicting at 3-, 5-, and 8-year CSS. OS: overall survival; CSS: cancer-specific survival. [file 12885_2020_7396_MOESM3_ESM.tif]
